# Qualität der frühzeitigen Sepsisdiagnostik auf deutschen Intensivstationen – Ergebnisse einer bundesweiten Umfrage

**DOI:** 10.1007/s00101-025-01562-1

**Published:** 2025-07-23

**Authors:** Stefanie Michel, Kristina Renckhoff, Florian Espeter, Daniel Richter, Markus A. Weigand, Tobias Schürholz, Thorsten Brenner, Simon Dubler

**Affiliations:** 1 Klinik für Anästhesiologie und Intensivmedizin, Universitätsklinikum Essen, Universität Duisburg-Essen, 45147 Essen, Deutschland; 2 Klinik für Anästhesiologie, Medizinische Fakultät der Universität Heidelberg, 69120 Heidelberg, Deutschland; 3 Sektion Systemische Inflammation und Sepsis (SIS) der Deutschen Interdisziplinären Vereinigung für Intensiv- und Notfallmedizin (DIVI) e. V., Berlin, Deutschland; 4 Klinik für Intensivmedizin und Intermediate Care, Universitätsklinikum RWTH Aachen, Aachen, Deutschland

**Keywords:** Sepsisdiagnostik, Sepsistherapie, Molekulardiagnostik, SOFA, qSOFA, Sepsisdiagnostic, Sepsistherapy, Moleculardiagnostic, SOFA, qSOFA

## Abstract

**Hintergrund:**

Die Versorgung von Sepsispatient*innen stellt eine anhaltende intensivmedizinische Herausforderung dar und führt selbst in medizinisch hochentwickelten Ländern zu inakzeptabel hohen Morbiditäts- und Mortalitätsraten.

**Fragestellung:**

Wie steht es um die Qualität der frühzeitigen Sepsisversorgung auf deutschen Intensivstationen?

**Material und Methoden:**

Zur Bewertung der Qualität der frühen Sepsisbehandlung führten wir eine nationale, internetbasierte Umfrage unter den Mitgliedern der Deutschen Interdisziplinären Vereinigung für Intensiv- und Notfallmedizin (DIVI) durch.

**Ergebnisse:**

Insgesamt wurden 1935 Intensivmediziner*innen in ganz Deutschland befragt, von denen 459 erfahrene Fachärzt*innen geantwortet haben – darunter 38 % Chefärzt*innen und 61 % Oberärzt*innen. Insgesamt berichteten 66 % der Teilnehmenden von der Anwendung von Screeninginstrumenten zur frühen Sepsiserkennung, wobei der qSOFA mit 71 % am häufigsten eingesetzt wurde. In 32,7 % der befragten Intensivstationen stand ein mikrobiologischer Dienst rund um die Uhr zur Verfügung. Interdisziplinäre Visiten unter Einbeziehung der Mikrobiologie oder der klinischen Pharmakologie fanden in 55,5 % bzw. 52,5 % der Intensivstationen statt. Molekulare diagnostische Verfahren (z. B. SeptiFast® (Roche, Basel, Schweiz)) zur schnellen Erregeridentifikation waren lediglich in 10,9 % der befragten Institutionen verfügbar.

**Diskussion:**

Zusammenfassend zeigt sich, dass der Einsatz des qSOFA-Scores – trotz abweichender aktueller internationaler Leitlinienempfehlungen – weiterhin sehr verbreitet ist. Zudem fanden interdisziplinäre Visiten mit der Mikrobiologie bzw. Pharmakologie nur auf der Hälfte der teilnehmenden Intensivstationen statt. Molekularbiologische diagnostische Verfahren zählen nach wie vor nur in wenigen Krankenhäusern zum diagnostischen Standard.

**Zusatzmaterial online:**

Zusätzliche Informationen sind in der Online-Version dieses Artikels (10.1007/s00101-025-01562-1) enthalten.

## Abstract (en)

**Background:**

The management of sepsis patients remains an ongoing challenge in intensive care medicine and leads to unacceptably high morbidity and mortality rates, even in medically highly developed countries.

**Objective:**

What is the state of the quality of early sepsis treatment on German intensive care units (ICUs)?

**Material and methods:**

To assess the quality of early sepsis treatment, we conducted a nationwide internet-based survey among members of the German Interdisciplinary Association of Critical Care and Emergency Medicine (DIVI).

**Results:**

A total of 1935 intensive care physicians across Germany were surveyed of whom 459 experienced specialist physicans responded, including 38% chief physicians and 61% senior physicians. Overall, 66% of participants reported the use of screening tools for early sepsis detection, with the quick-sepsis organ failure assessment (qSOFA) being the most frequently used (71%). In 32.7% of the surveyed ICUs a microbiologist was available around the clock. Interdisciplinary rounds involving a microbiologist or a clinical pharmacologist took place in 55.5% and 52.5% of ICUs, respectively. Molecular diagnostic procedures (e.g., SeptiFast®) for rapid pathogen identification were available in only 10.9% of the surveyed ICUs.

**Discussion:**

In summary, the use of the qSOFA score remains widespread despite deviating recommendations in current international guidelines. Additionally, interdisciplinary rounds involving microbiology or pharmacology were conducted in only half of the participating ICUs. Molecular biological diagnostic procedures are still not part of the diagnostic standard in most hospitals.

**Graphic abstract:**

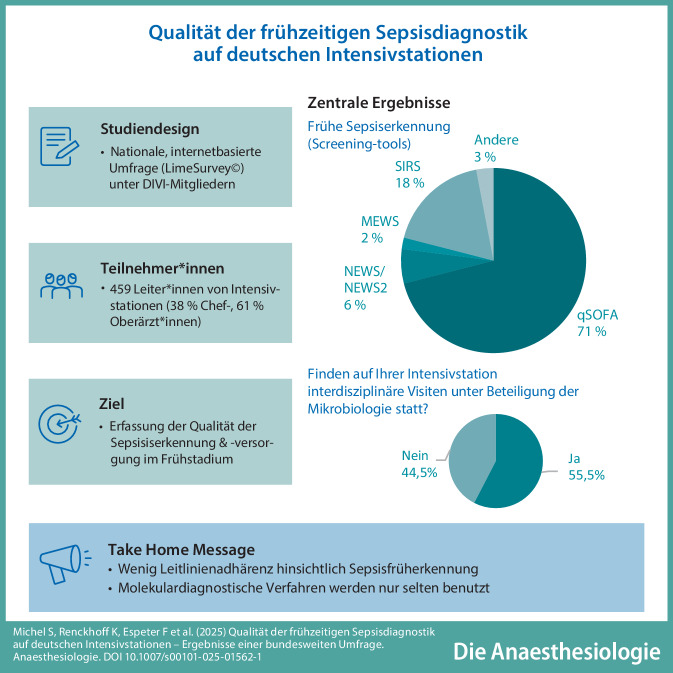

## Einführung

Sepsis ist eine der häufigsten Todesursachen in Deutschland – eine schnelle Diagnosestellung und ein zeitnaher Therapiebeginn sind lebensrettend. Unsere Umfrage zeigt: Trotz allen medizinischen Fortschritts besteht nach wie vor ein erheblicher Optimierungsbedarf in Bezug auf Sepsisscreening, Blutkulturmanagement und Antibiotikatherapie. Standardisierte Arbeitsanweisung (standard operating procedure (SOP)) und moderne Diagnostik könnten die Sepsisversorgung entscheidend verbessern. Das neue Qualitätssicherungsverfahren (QS) Sepsis des G‑BA (Gemeinsamer Bundesausschuss) ab 2026 bietet die Chance für eine nachhaltige Optimierung der Patient*innenenversorgung.

## Hintergrund

Die Sepsis ist definiert als lebensbedrohliche Organfunktionsstörung infolge einer fehlregulierten Wirtsreaktion auf eine Infektion [9]. Sepsis und septischer Schock zählen zu den gravierendsten Gesundheitsproblemen und fordern weltweit jährlich etwa 11 Mio. Menschenleben. Basierend auf der impliziten Definition der Sepsis als Infektion mit nachfolgendem Organversagen wird geschätzt, dass es in Deutschland pro Jahr rund 200.000 sepsisassoziierte Todesfälle gibt – dies entspricht etwa 47,2 % aller Krankenhaustodesfälle und 21,6 % aller Todesfälle im nationalen Vergleich [11].

Eine zeitgerechte Sepsisdiagnose sowie ein frühzeitiger Therapiebeginn sind von entscheidender Bedeutung für die Verbesserung der Überlebenschancen. Darüber hinaus können standardisierte Behandlungsabläufe („standard operating procedures“, SOP) in der Sepsisversorgung – etwa der frühzeitige und konsequente Einsatz von Screeninginstrumenten, Lactatmessungen, Blutkulturabnahmen sowie die rechtzeitige Verabreichung von Breitbandantibiotika, kristalloidem Volumenersatz und Katecholaminen – die Morbidität und Mortalität weiter senken.

Die Empfehlungen hinsichtlich der anzuwendenden Sepsis-Screening-Tools haben sich kürzlich geändert: Anstelle des quick-Sepsis Organ Failure Assessment (qSOFA) Score, der als alleiniges Screening-Tool nicht mehr empfohlen wird, werden nun die Kriterien des Systemic Inflammatory Response Syndrome (SIRS) sowie neue Scoring-Instrumente, u. a. der Modified Early Warning Score (MEWS) oder der National Early Warning Score (NEWS) empfohlen.

Die Messung der Lactatkonzentration ist ein Bestandteil des 1‑h-Sepsis-Bundles der Surviving Sepsis Campaign [35]. Wiederholte Lactatmessungen sind essenziell, da es eine gute Evidenz dafür gibt, dass es einen Zusammenhang zwischen hohen Lactatwerten und einem ungünstigen Verlauf bei kritisch kranken Patient*innen mit Verdacht auf Sepsis gibt [2, 9, 23]. Die Lactat-Clearance dient daher als wertvoller Parameter zur Steuerung der Volumentherapie, wobei Lactatwerte über 4 mmol/l auch bei Patient*innen mit Verdacht auf eine Sepsis ohne Schock einen negativen Prognosefaktor darstellen [6–8].

Die Entnahme von Blutkulturen vor der Gabe von Breitbandantibiotika ist ein weiterer wesentlicher Bestandteil der Sepsisdiagnostik. Bei Patient*innen mit Verdacht auf eine Sepsis wird die Abnahme von mindestens 2 oder mehr Blutkultursets (4 bis 6 Flaschen) empfohlen.

Neben der rechtzeitigen Gabe von Breitbandantibiotika ist auch eine rasche antiinfektive Deeskalation, sobald der klinische Verlauf und die mikrobiologischen Befunde dies zulassen, von großer Bedeutung. Deeskalationsstrategien sind wesentliche Elemente von Antibiotic-Stewardship(ABS)-Programmen, deren Umsetzung im klinischen Alltag zu einer verringerten Resistenzentwicklung, weniger antibiotikabedingten Nebenwirkungen und geringeren Gesamtkosten führt [27, 30]. Die direkten Effekte solcher Programme auf die Mortalitätsraten sind umstritten; so zeigte eine Vorher-nachher-Studie mit 1541 Intensivpatient*innen, dass eine restriktive Antibiotikatherapiestrategie (mit limitiertem Einsatz von Breitbandantibiotika) im Vergleich zu einer konventionellen Strategie (diese Strategie beinhaltete den Einsatz eines Breitbandantibiotikums als empirische Erstbehandlung im Falle einer Sepsis oder eines Infektionsverdachts, gefolgt von einer Deeskalation nach 48–72 h, basierend auf mikrobiologischen Daten) zu einer absoluten Reduktion der Mortalitätsrate um 6,1 % führte (*p* < 0,01) [21].

Um sowohl die Leitlinienadhärenz als auch die Sepsisprognose zu verbessern, veröffentlichte der Gemeinsame Bundesausschuss (G-BA) kürzlich eine Resolution zum Qualitätsmanagement in der Sepsisversorgung, die ab Anfang 2026 verpflichtend eingeführt werden soll [4]. Das Indikatorenset zur Risikoabschätzung auf Basis der der Machbarkeitsstudie „Diagnostik, Therapie und Nachsorge der Sepsis“ des IQTIG wird in Tab. 1 zusammengefasst. Ziel dieses Projekts war es, vor dem genannten Hintergrund mehr über den aktuellen Stand der Sepsisversorgung auf deutschen Intensivstationen zu erfahren. Zu diesem Zweck führten wir eine bundesweite Online-Umfrage unter den Mitgliedern der Deutschen Interdisziplinären Vereinigung für Intensiv- und Notfallmedizin (DIVI) durch.

**Tab. 1:** Messinstrumente und Qualitätsindikatoren der Sepsistherapie

Messinstrumente und Qualitätsindikatoren der Sepsistherapie
Blutkulturen vor Beginn der antimikrobiellen Therapie der Sepsis
Erfassung der Krankenhausletalität nach Sepsis
Multimodales Programm zur Prävention von Infektionen, die mit liegenden zentralen Venenkathetern assoziiert sind, zur Prävention von Sepsis im Krankenhaus
Therapieleitlinie zur antiinfektiven Therapie, unterstützt durch ein multidisziplinäres Antibiotic-Stewardship-Team
Verfügbarkeit einer Arbeitsanweisung (SOP) zur Versorgung bei Sepsis
Regelmäßige Schulungen zu Erkennung, Risikoeinstufung und Therapie von Sepsis
Erhöhung des Pflegegrads nach Sepsis innerhalb von 60 Tagen nach Entlassung

Adaptiert nach Qualitätssicherungsverfahren „Diagnostik, Therapie und Nachsorge der Sepsis“ des IQTIG [12]

*SOP* „standard operating procedure“

## Methoden

Zur Evaluierung der Qualität der Sepsisversorgung auf deutschen Intensivstationen wurde eine offene, internetbasierte Umfrage mit 58 Fragen unter Verwendung der LimeSurvey®-Applikation konzipiert. Die Umfrage wurde an 1935 medizinische Leiter*innen von Intensivstationen in Deutschland versandt, die alle Mitglieder*innen der Deutschen Interdisziplinären Vereinigung für Intensiv- und Notfallmedizin (DIVI) sind. Dies führte zu Antworten aus einer heterogenen Gruppe verschiedener medizinischer Fachrichtungen (z. B. Innere Medizin, Anästhesiologie, Neurochirurgie, Viszeralchirurgie etc.). Der Untersuchungszeitraum erstreckte sich von August bis September 2023. Jeder Fragebogen wurde ausschließlich an eine(n) Vertreter*in versendet, sodass eine doppelte Datenerhebung ausgeschlossen ist.

Die Umfrage umfasste Fragen zu den individuellen Qualifikationen der Teilnehmer*innen, detaillierte Angaben zum medizinischen Hintergrund sowie zur Infrastruktur der jeweiligen Intensivstation. Weiter wurden Fragen zu den Konzepten der Sepsisversorgung wie z. B. Sepsisscreening, mikrobiologische Diagnostik und Sepsisbehandlungen abgefragt. Alle Fragen, inkl. der entsprechenden Antwortmöglichkeiten, sind im Zusatzmaterial online: Tabelle S1 abrufbar. Da die Beantwortung einzelner Fragen unabhängig voneinander erfolgen konnte, wichen die Anzahl der Antworten sowie weitere statistische Kennwerte zwischen den Fragen voneinander ab. Für die Berechnung der Konfidenzintervalle der Proportionen wurde das Wilson-Score-Intervall verwendet. Der Z‑Wert für ein 95 %-Konfidenzintervall wurde auf 1,96 gesetzt. Daten wurden als Prozent positiver Antworten von allen Antworten und 95 % Konfidenzintervall (95 %-KI) dargestellt. Dichotome Variablen wurden mittels Chi^2^-Test analysiert. Tortendiagramme wurden mithilfe von GraphPad Prism (Version 10.2.3, GraphPad Software, La Jolla, USA) erstellt.

## Ergebnisse

### Qualifikationen der Befragten und Infrastruktur der Intensivstationen

Die Umfrage wurde an 1935 leitende Mediziner*innen von Intensivstationen in ganz Deutschland versandt und erreichte somit eine hochqualifizierte Gruppe von Teilnehmenden, bestehend aus Chefärzt*innen (38 %) sowie (leitenden) Oberärzt*innen (61 %). Von 366 zurückgesendeten Fragebogen wurden 93 vollständig ausgefüllt. Die übrigen 273 enthielten eine oder mehrere fehlende Antworten, etwa durch bewusstes Überspringen einzelner Fragen oder die Auswahl neutraler Optionen (z. B. „keine Antwort“ oder „Sonstiges“). Alle vorliegenden und für den jeweiligen Indikator ausreichend beantworteten Fragebogen wurden in die Analyse einbezogen. Obwohl die Umfrage gezielt an Intensivmediziner*innen aus sämtlichen Regionen Deutschlands gerichtet war, ließ sich eine auffallend hohe Beteiligung aus Bayern (25 %) und Nordrhein-Westfalen (15 %) verzeichnen. Der Großteil der befragten Mediziner*innen war im Fachbereich der Anästhesie tätig (83 %), wobei die behandelten Patient*innen nahezu alle medizinischen Fachrichtungen umfassten.

Die Ergebnisse der Umfrage zeigten, dass 46,2 % der teilnehmenden Intensivstationen jährlich zwischen 25 und 100 Patient*innen mit Sepsis oder septischem Schock behandelten, während 30,8 % der Stationen zwischen 101 und 250 septische Patient*innen pro Jahr versorgten. Nahezu 80 % der Befragten, die den Fragebogen vollständig ausfüllten, gaben an, über eine Standardarbeitsanweisung (SOP) für die Behandlung von Patient*innen mit Sepsis und septischem Schock zu verfügen. Ein Patient*innenendatenmanagementsystem (PDMS) war in 47,9 % der erfassten Intensivstationen verfügbar.

### Screeninginstrumente zur Diagnose von Sepsis und septischem Schock

**Abb. 1:**
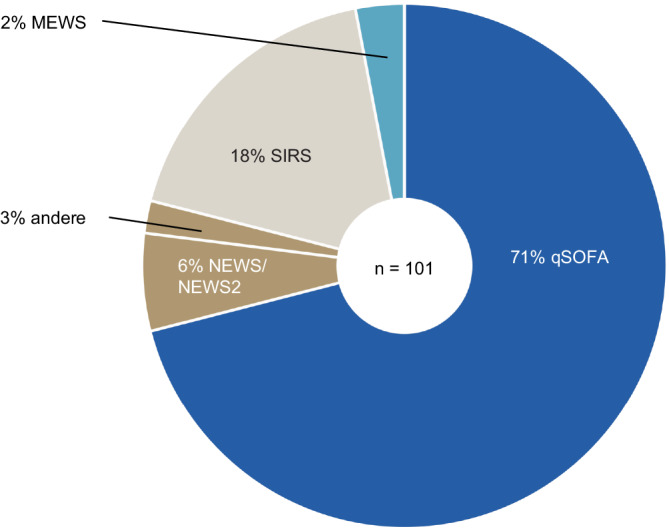
Häufigste verwendete Instrumente zum Sepsisscreening. *qSOFA* quick Sequential Organ Failure Assessment, *NEWS* Early Warning Score, *MEWS* Modified Early Warning Score, *SIRS* systemisches inflammatorisches Response-Syndrom

Screeninginstrumente zur Diagnose einer Sepsis wurden in 66 % der teilnehmenden Intensivstationen eingesetzt, wobei der qSOFA-Score mit 71 % das am häufigsten verwendete Instrument darstellte. Die SIRS-Kriterien wurden von 18 % der Intensivstationen genutzt, während der MEWS-Score und der NEWS/NEWS2-Score lediglich in 2 % bzw. 6 % der Stationen Anwendung fanden (Abb. 1). Insgesamt wurde der Sequential Organ Failure Assessment Score (SOFA-Score) zur Diagnostik der Sepsis in 51 % der erfassten Intensivstationen mindestens einmal täglich berechnet. Diese Berechnung erfolgte überwiegend manuell (47 %), während sie in 22 % der Fälle assistiert und in 20 % vollautomatisch durchgeführt wurde. Die Bestimmung des Lactatwerts als diagnostisches Kriterium für einen septischen Schock wurde in 83 % der befragten Intensivstationen vorgenommen. Seit der Publikation der SEPSIS-3-Definition im Jahr 2016 haben 57,6 % der befragten Intensivstationen ihre Diagnostik zur Sepsis angepasst. Dabei wurden insbesondere die Schulungsmaßnahmen (47,8 %) sowie die Standardarbeitsanweisungen zur Sepsisversorgung (41,3 %) aktualisiert.

### Mikrobiologische Diagnostik und Management bei Patient*innen mit Sepsis oder septischem Schock

Lediglich 32,7 % der erfassten Intensivstationen verfügten über eine rund um die Uhr verfügbare mikrobiologische Expertise. Interdisziplinäre Visiten mit der Mikrobiologie oder der klinischen Pharmakologie fanden in 55,5 % (mit einem Median von 1,4 Visiten/Woche) bzw. 52,5 % (mit einem Median von 1,1 Visiten/Woche) der Intensivstationen statt. Interdisziplinäre Visiten durch die Krankenhaushygiene mit dem Ziel von Ausbildung von Personal, Kontrolle von Maßnahmen und fallspezifischer Beurteilung wurden als „Hygieneteam“ abgefragt. Solche Visiten waren im Stationsalltag weniger präsent und besuchten die Intensivstationen lediglich einmal pro Monat in 64 % der Fälle. Hinsichtlich des Blutkulturmanagements wurden an den jeweiligen Standorten durchschnittlich 2,6 ± 1,3 Blutkulturflaschen aus 1,8 ± 0,5 unterschiedlichen Entnahmestellen abgenommen. Dies resultierte in einer Entnahmerate von 135,6 Blutkulturen/1000 Patient*innentagen (SA =± 130,7). Die labordiagnostische Verarbeitung der Blutkulturen erfolgte in 68 % der Fälle rund um die Uhr und in 89 % der Fälle an 7 Tagen/Woche. Eine SOP für Blutkulturen war lediglich in 68 % der befragten Intensivstationen etabliert.

Nur 10 % der Intensivstationen verfügten über ein eigenes Blutkulturinkubationssystem direkt auf der Station. Die Transportlogistik variierte erheblich zwischen den einzelnen Einrichtungen: Während 50 % der Blutkulturen innerhalb von 2 h ins mikrobiologische Labor gelangten, erreichten 21,8 % der Proben das Labor innerhalb von 6 h. In 16,8 % der Fälle dauerte der Transport maximal 12 h, während er in 8,9 % der Einrichtungen sogar mehr als 12 h in Anspruch nahm. In 57,4 % der Intensivstationen wurden die Parameter „time-to-positivity“ sowie die minimale Hemmkonzentration (MHK) berichtet (63 %). Vorläufige Antibiogramme wurden in 73,9 % der befragten Krankenhäuser erstellt. Moderne, nichtkulturbasierte molekularbiologische Methoden zur schnellen Pathogenidentifikation, wie beispielsweise SeptiFast®, wurden hingegen nur in 10,9 % der Einrichtungen genutzt.

Präventive Maßnahmen, wie der routinemäßige Wechsel von Kathetern, wurden in 24 % der Intensivstationen umgesetzt. Auf Basis der erhobenen Daten ließ sich jedoch weder die mediane Verweildauer der Katheter noch das Vorgehen bei laborchemischen und/oder klinischen Hinweisen auf eine Infektion eindeutig erfassen. Hingegen war präventives Hygienetraining weit verbreitet und wurde in 95 % der Einrichtungen mindestens einmal jährlich (98 %) durchgeführt.

### Nationale Kooperation und Surveillance

Insgesamt nahmen 82 % der Krankenhäuser, in denen die Befragten tätig waren, an den Krankenhaus-Infektions-Surveillance-System(KISS)-Modulen des Nationalen Referenzzentrums für Surveillance von nosokomialen Infektionen teil, wobei das ITS-KISS-Modul mit 32,7 % am häufigsten genutzt wurde. Unabhängig von der nationalen Surveillance führten nahezu alle teilnehmenden Institutionen eine routinemäßige Überwachung nosokomialer Infektionen (97 %) durch und pflegten Statistiken zur Antibiotikaresistenz (96 %). In der Regel oblag die Verantwortung für diese Überwachung der medizinischen Leitung der jeweiligen Intensivstationen (86 %), wobei mindestens einmal jährlich eine Rückmeldung über die erhobenen Daten erfolgte (46 %).

Die Ergebnisse weiterer, bisher nicht abgebildeter Fragen sind in Tab. 2 dargestellt. Eine vollständige Übersicht aller ursprünglichen Fragen der Umfrage sowie der möglichen Antwortoptionen ist im Zusatzmaterial online: Tabelle S1 enthalten.

**Tab. 2:** Ausgewählte Fragen und Antwortmöglichkeiten aus der Originalumfrage mit LimeSurvey®

Fragen	Antwortmöglichkeiten	Prozent (%), Mittelwert ± Standardabweichung (SA)
Q1: Welche Qualifikation besitzen Sie? (*n* = 100)	Oberarzt	*24* *% *(16,7–31,3 %)
	Leitender Oberarzt	*38* *% *(28,5–47,5 %)
	Chefarzt	*38* *% *(28,5–47,5 %)
Q3: Bitte geben Sie die Anzahl der Intensivbetten an, für die Ihre Abteilung verantwortlich ist? (*n* = 125)	Mittelwert ± SA	*13* *±* *13*
Q4: Wie viele Betten umfasst Ihr Krankenhaus insgesamt? (*n* = 126)	< 100	*2* *% *(0,7–5,8 %)
	100–250	*26* *% *(19,2–34,1 %)
	251–500	*25* *% *(18,4–33,0 %)
	501–1000	*33* *% *(25,3–41,6 %)
	> 1000	*14* *% *(9,1–21,0 %)
Q5: Welche Fachrichtung ist für die Leitung Ihrer Intensivstation verantwortlich? (*n* = 88)	Anästhesiologie	*83* *% *(74,0–89,3 %)
	Kardiologie	*12* *% *(6,5–21,3 %)
	Pneumologie	*3* *% *(1,0–9,3 %)
	Neurologie	*1* *% *(0,2–6,2 %)
	Neurochirurgie	*1* *% *(0,2–6,2 %)
Q6. Aus welchen medizinischen Fachrichtungen behandeln Sie Patientinnen und Patienten? (*n* = 704, Mehrfachantworten möglich)	Herzchirurgie	*2* *% *(1,2–3,5 %)
	Neurochirurgie	*5* *% *(3,6–7,0 %)
	Hämatologie/Onkologie	*8* *% *(6,2–10,5 %)
	Anästhesiologie	*12* *% *(9,7–14,7 %)
	Pneumologie	*12* *% *(9,7–14,7 %)
	Kardiologie	*13* *% *(10,5–15,9 %)
	Gastroenterologie	*13* *% *(10,5–15,9 %)
	Allgemeinchirurgie	*13* *% *(10,5–15,9 %)
	Orthopädie/Unfallchirurgie	*13* *% *(10,5–15,9 %)
Q9: Wie hoch ist der Anteil der maschinell beatmeten Patientinnen und Patienten auf Ihrer Intensivstation? (*n* = 120)	Mittelwert ± SA	*30* *%* *±* *17* *%*
Q10: Wie lange beträgt die durchschnittliche Verweildauer auf Ihrer Intensivstation? (In Tagen, *n* = 115)	Mittelwert ± SA	*6* *±* *4*
Q11: Wie ist das ärztliche Betreuungsverhältnis auf Ihrer Intensivstation während Bereitschaftsdiensten (nach 16:00 Uhr oder an Wochenenden)? (*n* = 119)	Mittelwert ± SA	*8* *±* *4*
Q12: Wie hoch ist der Anteil an Fachärztinnen und Fachärzten in Ihrem intensivmedizinischen Team? (*n* = 118)	Mittelwert ± SA	*46* *%* *±* *27* *%*
Q14: Wie ist das pflegerische Betreuungsverhältnis auf Ihrer Intensivstation? (*n* = 117)	Mittelwert ± SA	*3* *±* *3*
Q15: Wie hoch ist der Anteil an spezialisierten Pflegekräften in Ihrem Pflegeteam? (*n* = 116)	Mittelwert ± SA	*46* *%* *±* *18* *%*
Q16: Berücksichtigen Sie bei der Diagnose „septischer Schock“ gemäß SEPSIS‑3 den Lactatspiegel des Patienten? (*n* = 118)	Ja	*91,5* *% *(85,7–95,3 %)
	Nein	*8,5* *% *(4,7–14,3 %)
		*p* *<* *0,001*
Q17: Gab es seit der Publikation der SEPSIS-3-Definition im Januar 2016 diesbezügliche Veränderungen in Ihrer Abteilung? (*n* = 117)	Ja	*56,4* *% *(47,4–65,0 %)
	Nein	*43,6* *% *(35,0–52,6 %)
		*p* *=* *0,165*
Q18: Wenn ja, welche?	SOP	*42,1* *% *(33,3–52,1 %)
	Schulung	*51,4* *% *(42,7–59,6 %)
Q19: Bei einem Patienten mit Sepsis oder septischem Schock: Wie viele routinemäßige Laboruntersuchungen (Blutgasanalysen ausgeschlossen) werden täglich auf Ihrer Intensivstation durchgeführt? (*n* = 117)	0	*1* *% *(0,2–5,3 %)
	1	*47* *% *(38,1–55,9 %)
	2	*31* *% *(23,3–39,9 %)
	3	*13* *% *(8,0–20,3 %)
	> 3	*8* *% *(4,2–14,8 %)
Q19.1: Welche dieser Laborparameter, als Bestandteil des SOFA-Scores, sind Teil Ihrer routinemäßigen Laboruntersuchungen? (Mehrfachantworten möglich, *n* = 117)	Kreatinin	*100* *% *(96,9–100 %)
	Thrombozyten	*100* *% *(96,9–100 %)
	Bilirubin	*87* *% *(79,6–92,1 %)
Q19.2: Wird routinemäßig eine arterielle Kanüle bei allen Patientinnen und Patienten mit Verdacht auf Sepsis auf Ihrer Intensivstation gelegt? (*n* = 117)	Ja	*62* *% *(53,0–70,3 %)
	Nein	*38* *% *(29,7–47,0 %)
		*p* *=* *0,007*
Q19.3: Wie häufig wird der Horowitz-Index bei Patientinnen und Patienten mit Sepsis oder septischem Schock auf Ihrer Station täglich berechnet? (*n* = 117)	0	*14* *% *(8,8–21,6 %)
	1	*14* *% *(8,8–21,6 %)
	2	*10* *% *(5,8–17,1 %)
	3	*25* *% *(18,0–33,8 %)
	> 3	*37* *% *(28,9–46,0 %)
Q19.3.1: Wie erfolgt die Berechnung? (*n* = 101)	Automatisiert	*55* *% *(46,2–63,8 %)
	Manuell	*45* *% *(36,2–54,1 %)
		*p* *=* *0,273*

Die Antworten werden als Prozentwerte (%), Mittelwert ± Standardabweichung (SA) angegeben

*n* Anzahl der Teilnehmenden, die die jeweilige Frage beantwortet haben, *ICU* Intensive Care Unit, *SOFA* Sequential Organ Failure Assessment

## Diskussion

In dieser Studie präsentieren wir die Ergebnisse einer deutschlandweiten, onlinebasierten Umfrage, die mit Unterstützung der Deutschen Interdisziplinären Vereinigung für Intensiv- und Notfallmedizin (DIVI) durchgeführt wurde. Ziel der Untersuchung war die Erfassung der infrastrukturellen Gegebenheiten sowie der aktuellen Praxis in der Diagnostik, dem Screening und dem Management von Sepsis bzw. septischem Schock auf deutschen Intensivstationen.

Sepsis und septischer Schock werden nach wie vor zu spät und zu selten erkannt [26]. Alle 6 bis 7 Minuten verstirbt ein Mensch in Deutschland an einer Sepsis [1]. Eine frühzeitige Erkennung der Erkrankung sowie die rasche Identifikation des auslösenden Erregers sind essenziell, um die Überlebensrate der Patient*innen zu verbessern [24]. Trotz kontinuierlicher medizinischer Fortschritte zeigt eine systematische Analyse der 30-Tage-Sterblichkeit bei septischem Schock über die letzten 10 Jahre eine weiterhin hohe Mortalitätsrate von 32,5 % (95 %-KI: 31,7–33,3) [1, 10]. Auch auf internationaler Ebene zeigen sich Defizite in der Sepsisversorgung: Eine multinationale Querschnittstudie von Scheer et al. (2025) identifizierte auf Basis von Daten aus 69 Ländern erhebliche Unterschiede in Diagnostik, Therapie und Organisation sepsisbezogener Maßnahmen zwischen europäischen Krankenhäusern [33]. Besonders deutlich waren die Defizite in der strukturierten Umsetzung von Früherkennung von Sepsis und standardisierten Behandlungswegen. Die Ergebnisse verdeutlichen den Bedarf an Standardisierung in der Sepsisversorgung und stützen die im Rahmen der aktuellen Studie erhobenen Befunde auf nationaler Ebene. Auf nationaler Ebene hat der G-BA im November 2023 ein Qualitätssicherungsverfahren zu Diagnostik, Behandlung und Nachsorge der Sepsis initiiert, um diesem Problem entgegenzuwirken [4]. Dieser Bericht soll potenzielle Optimierungsstrategien aufzeigen, um die stationäre Versorgung betroffener Patient*innen zu verbessern. In Zusammenarbeit mit einem Expertengremium hat der G‑BA ein gezielteres und datenoptimiertes Dokumentationsverfahren entwickelt. Dieses soll dem Gesetzgeber die Analyse der Behandlungsqualität in Krankenhäusern erleichtern. Letztlich dient es dazu, Handlungsbedarfe präziser zu identifizieren und die gesetzlich verankerte Qualitätssicherung in der Versorgung von Patient*innen mit Sepsis oder septischem Schock zu gewährleisten.

Diese Maßnahmen sind besonders wichtig, da die Implementierung standardisierter SOP für die Diagnose und Behandlung von Sepsis lediglich von 50 % der befragten Gesundheitseinrichtungen berichtet wurde, obschon der G‑BA das Vorhalten dieser ausdrücklich als Qualitätsindikator benennt. Interessanterweise wurden 83 % der teilnehmenden Intensivstationen von anästhesiologischen Abteilungen geleitet, was über dem in einer europäischen Erhebung berichteten Anteil von etwa 70 % liegt [39]. Unterschiede in dieser Verteilung könnten relevante, bislang nicht abschließend beurteilbare Effekte auf die erhobenen Indikatoren gehabt haben. Die vorliegende Umfrage richtete sich ausschließlich an das Leitungspersonal von Intensivstationen, sodass keine Daten aus anderen beteiligten Bereichen wie der präklinischen Versorgung, der Notaufnahme oder der normalstationären Versorgung erfasst wurden. Mit einer Bettenzahl > 500 bei 47 % der eingeschlossenen Zentren könnte eine Selektion von (Sub‑)Maximalversorgern eingetreten sein, wodurch die Übertragbarkeit der Ergebnisse eingeschränkt wird. Wie bei allen umfragebasierten Studien besteht auch hier das Risiko eines Reporting Bias (Verzerrung der Berichterstattung). Insbesondere ist nicht auszuschließen, dass bestimmte Angaben, etwa zur Struktur- oder Prozessqualität, systematisch überpositiv dargestellt wurden.

Eine retrospektive Studie aus dem US-Bundesstaat New York, veröffentlicht im Jahr 2019, zeigte, dass eine verpflichtende, protokollgestützte Sepsisversorgung mit einem stärkeren Rückgang der Sepsismortalität einherging als in Kontrollstaaten ohne entsprechende Regulierungen [16]. Dies steht im Einklang mit den aktuellen Leitlinien der *Surviving Sepsis Campaign*, welche die Implementierung von Leistungsverbesserungsprogrammen für Sepsis, einschließlich SOP, empfehlen [9]. Neben der geringen Akzeptanz von SOP gaben nur 66 % der Teilnehmenden an, ein strukturiertes Sepsisscreening durchzuführen. Häufig scheint dabei der qSOFA-Score (71 %) zum Einsatz zu kommen. Obwohl der qSOFA-Score weiterhin in den aktuellen Sepsis-3-Leitlinien enthalten ist, wird seine Anwendung zunehmend hinterfragt, da er in einigen Studien als unzureichend sensitiv für die frühe Sepsiserkennung gilt [29, 32]. Stattdessen werden die SIRS-Kriterien, der NEWS oder der *Modified Early Warning Score *(MEWS) für das Sepsisscreening empfohlen, da sie eine höhere Sensitivität aufweisen [6, 9]. Während die *Surviving Sepsis Campaign* die Verwendung des qSOFA-Score weiterhin nicht empfiehlt, wird keine spezifische Alternative für das Screening mit Hinweis auf die individuellen Einschränkung von Scoring-Systemen vorgeschlagen [28, 35]. Ein weiterer zentraler Aspekt des Sepsis-Managements ist die Zeit bis zur Diagnose, da jede Verzögerung in der Antibiotikatherapie mit einer erhöhten Mortalität assoziiert ist [19]. Dennoch berichteten lediglich 57 % der Intensivmediziner*innen über die tägliche Berechnung des SOFA-Scores auf ihrer Intensivstation. Unzureichende oder lückenhafte Umsetzung, die verzögerte Abbildung kritischer Veränderungen durch den Score oder insuffizienter intra- und interprofessioneller Transfer können zu einer erheblichen diagnostischen Zeitlücke bei der frühen Erkennung von Sepsis führen [29, 34]. Die vorliegende Studie identifiziert eine Lücke bei der initialen Lactatbestimmung (17 %) sowie das Fehlen einer standardisierten SOP zur Sepsisbehandlung in 20 % der Intensivstationen. Internationale Leitlinien wie die Surviving Sepsis Campaign empfehlen die Messung des Lactats innerhalb der ersten Stunde sowie eine wiederholte Bestimmung bei initial erhöhten Werten zur Verlaufsbeurteilung und zur Steuerung der Therapie. Das Fehlen dieser Maßnahmen auf einem relevanten Anteil der Intensivstationen könnte strukturelle oder prozessuale Ursachen haben und erscheint auch im internationalen Vergleich auffällig [5, 9, 22].

Nur 63,7 % der Intensivmediziner*innen hatten Blutkulturen in ihr diagnostisches Vorgehen integriert. Laut Umfrage wurden durchschnittlich 135,6 Blutkulturflaschen/1000 Patient*innentagen entnommen (SD 130,7). Gemäß der aktuellen Leitlinien sollten vor Beginn einer neuen antimikrobiellen Therapie bei Verdacht auf Sepsis mindestens 2 Sätze aerober und anaerober Blutkulturen abgenommen werden [9]. Ein Blick über nationale Grenzen hinaus zeigt dabei deutliche Unterschiede: Eine europaweite Erhebung des *European Antimicrobial Resistance Surveillance Network* ergab, dass Deutschland hinsichtlich der Häufigkeit von Blutkulturen nur im unteren Drittel liegt [17]. Während in Ländern wie Belgien, Dänemark, Finnland, Portugal und Spanien über 100 Blutkulturen/1000 Patient*innentage Standard waren, lag der Wert in Deutschland lediglich bei 16,6 [17]. Die aktuelle Erhebung zeigt hier jedoch eine deutliche Verbesserung: Die Anzahl der entnommenen Blutkulturen in den teilnehmenden Krankenhäusern war mehr als 8‑mal so hoch wie in der vorherigen Studie [17].

Die kulturbasierte Pathogendiagnostik bleibt weiterhin der Goldstandard, ist jedoch zeitaufwendig und weist eine geringe Nachweisrate auf, was die zielgerichtete antibiotische Therapie erschwert [7]. In der vorliegenden Umfrage gaben nur 10 % der Intensivstationen an, über ein eigenes Blutkulturinkubationssystem auf der Station zu verfügen. Die Transportlogistik mikrobiologischer Proben, einschließlich der Blutkulturen, stellt in Deutschland ein erhebliches strukturelles Problem dar, das die Diagnostik und damit die Therapie von Blutstrominfektionen maßgeblich beeinflussen kann [31]. Vor diesem Hintergrund ist die „time to positivity“ ein relevanter Parameter, dessen Aussagekraft durch Transport- und Bearbeitungszeiten innerhalb und außerhalb der Station erheblich negativ beeinflusst werden kann [8]. Schnellere, kulturunabhängige molekulardiagnostische Verfahren könnten vielversprechende Alternativen darstellen. Besonders die plasmabasierte Detektion zirkulierender zellfreier DNA mittels *Next Generation Sequencing* hat sich bereits als vielversprechend für die Identifikation von Pathogenen bei Blutstrominfektionen erwiesen [13]. Aktuell liegt, abgesehen von bestimmten spezifischen Fragestellungen, keine ausreichende Evidenz für den generellen Einsatz kulturunabhängiger Verfahren bei Blutstrominfektionen vor. Fortlaufende wissenschaftliche Bemühungen könnten jedoch künftig die verfügbare Evidenz wesentlich erweitern [3]. In der vorliegenden Umfrage gaben nur 11 % der Intensivstationen an, molekulardiagnostische Methoden zu nutzen, wobei die PCR-basierte Methode SeptiFast® am häufigsten genannt wurde. Die initiale kalkulierte Antibiotikatherapie wurde nicht erfasst.

Neben der Anzahl der entnommenen Blutkulturen variierte auch die Transportzeit der Proben zum Labor erheblich. Insgesamt waren die Transportzeiten in der Umfrage jedoch erfreulich kurz: In über 70 % der teilnehmenden Intensivstationen lagen sie unter 6 h. Dies deutet darauf hin, dass in den vergangenen 10 Jahren gezielt in die entsprechende Infrastruktur investiert wurde. Eine 2013 veröffentlichte Studie ergab, dass die Transportzeit für Blutkulturen in Deutschland im Durchschnitt 13,3 h betrug, verglichen mit 10,4 h in den Niederlanden und nur 9 h in Schweden. Solche Verzögerungen beeinflussen die mikrobiologischen Ergebnisse erheblich: Längere Transportzeiten führen zu einer verzögerten Identifikation von Erregern und somit zu einer verspäteten Anpassung der antibiotischen Therapie. Dies kann sich negativ auf den Behandlungserfolg auswirken, die Kosten erhöhen und die Rate resistenter Bakterien steigern [31].

Deeskalationsstrategien sind essenzielle Bestandteile von *Antimicrobial-Stewardship*(ABS)-Programmen. Ihre Implementierung reduziert die Resistenzentwicklung von Mikroorganismen, Nebenwirkungen und die Behandlungskosten [27, 30]. Dennoch gab nur etwa die Hälfte der Befragten an, mindestens einmal wöchentlich eine Visite mit einem Mikrobiologen durchzuführen; dies galt ebenso für regelmäßige Visiten mit einem klinischen Pharmakologen. Diese Werte sind niedriger als die aus anderen europäischen Krankenhäusern: Eine 2021 durchgeführte Erhebung in 812 europäischen Krankenhäusern ergab, dass 63 % über ein ABS-Team verfügen [20]. In der globalen TEAMICU-Studie, die 812 Intensivstationen weltweit umfasste, waren Infektiologen in 67 % der Krankenhäuser für Konsultationen verfügbar, während weitere 16 % externe Spezialisten hinzuziehen konnten. Zudem standen in 60 % der Krankenhäuser klinische Mikrobiologen für Inhouse-Konsultationen zur Verfügung, während 22 % externe Beratungen nutzten [20]. Eine kanadische Studie zeigte, dass Pharmakolog*innen in 85 % der Intensivstationen regelmäßig anwesend waren – ein Wert, der deutlich über den Ergebnissen der vorliegenden Umfrage liegt [15].

Die Einführung eines Patient*innenendatenmanagementystems (PDMS) ist nicht nur für die automatisierte Dokumentation komplexer intensivmedizinischer Fälle von Bedeutung, sondern auch für das Potenzial von durch künstliche Intelligenz (KI) gestützten Diagnosen und die frühzeitige Erkennung von Sepsis. Bereits drei Studien belegen eine signifikante Reduktion der sepsisassoziierten Mortalität nach Implementierung eines PDMS in der Intensivmedizin [36–38]. In der aktuellen Erhebung berichteten 47,7 % der Teilnehmenden, dass in ihrer Intensivstation ein PDMS zur Verfügung steht – ein Wert, der dem *VIS-ITS Survey* von 2017 entspricht, in dem lediglich 31 % der deutschen Intensivstationen ein PDMS zur Dokumentation verwendeten [14]. Im internationalen Vergleich ist dieser Anteil jedoch weiterhin gering. In der *TEAMICU*-Studie nutzten 59 % der 812 befragten Intensivstationen ein elektronisches PDMS [20]. Zahlreiche Studien haben die Vorteile eines PDMS in der Intensivmedizin bereits belegt, darunter eine Verringerung von Medikationsfehlern und unerwünschten Nebenwirkungen, eine Zeitersparnis in der Dokumentation sowie eine höhere Zufriedenheit des medizinischen Personals [36–38]. Ein PDMS sollte eine schnelle, systematische und umfassende Visualisierung aller relevanten Patient*innendaten ermöglichen, einschließlich Laborbefunden, Bildgebung, EKG und mehr. Zudem sollten Warnhinweise für auffällige Werte sowie automatische Sicherheitsmechanismen zur Arzneimittelverordnung integriert sein. Künftig könnten PDMS auch maschinelles Lernen und künstliche Intelligenz einbinden [18, 25]. Deutschland hat in der Digitalisierung seiner Krankenhäuser allerdings noch erheblichen Investitionsbedarf, weshalb der G-BA im Jahr 2023 einen entsprechenden Beschluss formulierte [4]. Solange eine internationale Standardisierung der Sepsisdiagnostik, -behandlung und -versorgung ausbleibt, bleibt der Vergleich zwischen Einrichtungen oder Ländern schwierig. Zudem konzentrieren sich bestehende Sepsisstudien überwiegend auf patient*innenspezifische Faktoren, während die Auswirkungen unterschiedlicher Krankenhausstrukturen auf die Behandlungsergebnisse oft unberücksichtigt bleiben. Eine internationale Schnittstelle zu Aggregation und wissenschaftlicher Analyse von Gesundheitsdaten könnte einen entscheidenden Fortschritt in der Sepsisversorgung darstellen. Die umfassende Integration von PDMS in die klinische Routine der deutschen Intensivstationen bietet dabei eine bislang ungenutzte Chance für eine schnellere und präzisere Sepsisdiagnostik.

## Fazit für die Praxis

Zur Verbesserung der Sepsisdiagnostik und -therapie ist insbesondere die konsequente Einhaltung nationaler und internationaler Leitlinien essenziell. Die Etablierung klinikweiter, automatisierter Algorithmen zur Sepsisfrüherkennung sowie die Implementierung standardisierter Abläufe (SOP) zur Diagnostik und zur Therapie könnten die Versorgungsqualität weiter steigern. Die interdisziplinäre Zusammenarbeit stellt dabei einen zentralen Baustein dar, um eine strukturierte, evidenzbasierte und patientenorientierte Versorgung zu gewährleisten.

## Supplementary Information

Tab. S1: Alle Fragen und mögliche Antworten in dem Survey

## Data Availability

Die in dieser Studie erhobenen Datensätze können auf begründete Anfrage beim Korrespondenzautor angefordert werden.
