# Supplementary material for: Quality of early sepsis diagnostics in German intensive care units—Results of a nationwide survey
Source: Anaesthesiologie. 2025 Jul 23;74(8):489–99. [Article in German] doi: 10.1007/s00101-025-01562-1 (PMC12313789; doi:10.1007/s00101-025-01562-1)
Supplement: Supplementary file 1 — Tab. S1: Alle Fragen und mögliche Antworten in dem Survey [file 101_2025_1562_MOESM1_ESM.pdf]

Zusatzmaterial zum Beitrag *„Qualität der frühzeitigen Sepsisdiagnostik auf deutschen Intensivstationen – Ergebnisse einer bundesweiten Umfrage“* von Michel S, Renckhoff K, Espeter F et al. (2025) in *Die Anaesthesiologie*.
Beitrag und Zusatzmaterial stehen Ihnen auf www.springermedizin.de zur Verfügung. Bitte geben Sie dort den Beitragstitel in die Suche ein.

Online supporting information

Questions	Possible answers
Q1: What is your qualification?	*Assistant doctor*<br>*Specialist*<br>*Senior physician*<br>*Senior consultant*<br>*Chief physician*
Q2: In which German state is your hospital located?	*Baden – Württemberg*<br>*Bavaria*<br>*Berlin*<br>*Brandenburg*<br>*Bremen*<br>*Hamburg*<br>*Hesse*<br>*Mecklenburg – Western Pomerania*<br>*Lower Saxony*<br>*North Rhine – Westphalia*<br>*Rhineland – Palatinate*<br>*Saarland*<br>*Saxony*<br>*Saxony – Anhalt*<br>*Schleswig – Holstein*<br>*Thuringia*
Q3: Please indicate the number of intensive care beds for which your department is responsible (only respiratory beds, no intermediate care)?	*Number of beds*
Q4: How many hospital beds does your hospital have in total?	*Number of beds*
Q5: Which specialist discipline is responsible for managing your intensive care unit?	*Anesthesiology*<br>*General and visceral surgery*<br>*Neurosurgery*<br>*Traumatology/Orthopaedics*<br>*Cardiac surgery*<br>*Pulmonology*<br>*Cardiology*<br>*Gastroenterology and Hepatology*<br>*Hemato – oncology*<br>*Pediatrics*<br>*Neurology*<br>*other*<br>*No answer*
Q6. Which specialist disciplines do you treat patients from?	*Anesthesia*<br>*General and visceral surgeryNeurosurgery*<br>*Traumatology/Orthopaedics*<br>*Cardiac surgery*<br>*Pulmonology*

Q7: Which medical disciplines are involved in your ICU?	Cardiology<br>Gastroenterology and Hepatology<br>Haemato – oncology<br>Pediatrics<br>Neurology<br>Other<br>Anesthesia<br>General and visceral surgeryNeurosurgery<br>Traumatology/Orthopaedics<br>Cardiac surgery<br>Pulmonology<br>Cardiology<br>Gastroenterology and Hepatology<br>Haemato – oncology<br>Pediatrics<br>Neurology<br>Other
Q8. How many patients with sepsis / septic shock are treated in your department each year?	< 25<br>$25 - 100$<br>$101 - 250$<br>$251 - 400$<br>> 401<br>no answer
Q9: How high is the proportion of invasively ventilated patients in your intensive care unit?	number x
Q10: What is the average length of stay in your intensive care unit?	number x
Q11: What is the medical staffing ratio on your intensive care unit when on duty (after 4 p.m. or at weekends)?	number x
Q12: How high is the proportion of specialists in your medical intensive care team?	number x
Q13: How many medical team members have the additional qualification "Special anaesthesiologic intensive care medicine"	number x
Q14: What is the nursing staffing ratio in your intensive care unit?	number x
Q15: How high is the proportion of specialist nursing in your nursing team?	number x
Q16: Do you carry out sepsis screening in the emergency department or on the normal wards of your facility? If yes, what tool?	SIRS<br>MEWS<br>NEWS<br>qSOFA<br>other

Q17: Do you take the patient's lactate level into account when diagnosing "septic shock" according to SEPSIS-3?	$number\ x$
Q18: Have there been any changes in your department in this regard since the SEPSIS-3 definition was published in January 2016? If yes, which one?	SOP<br>*Education*<br>*other*
Q19: For a patient in sepsis or septic shock: What is the minimum number of routine laboratory tests (not counting blood gas analyses (BGAs)) performed daily in your intensive care unit?	*1*<br>*2*<br>*3*<br>> 3<br>$other$<br>$no\ answer$
Q20: Which of these laboratory parameters, which are components of the SOFA score, are part of your routine laboratory?	*Creatinine*<br>*Bilirubine*<br>$Thrombocytes$<br>*other*
Q21: Is an arterial line routinely fitted to every patient treated with suspected sepsis in your ICU?	*Yes/No*
Q22: How many times a day is the Horowitz quotient calculated on your ward for a patient with sepsis or septic shock?	*0*<br>*1*<br>*2*<br>3<br>> 3<br>$other$
Q23: How is the calculation made?	$manually$<br>*automatically*<br>*no answer*
Q24: Has a patient data management system (PDMS) been introduced in your intensive care unit?	*Yes/No*
Q25: Do you currently calculate the SOFA score regularly (at least once a day)?	*Yes/No*
Q25-1: How is the calculation made?	$manually$<br>*automatically*<br>*no answer*
Q26: Is there a microbiologist on duty 24/7 in your hospital?	*Yes/No*
Q27: Do interdisciplinary rounds involving microbiology take place in your intensive care unit?	*Yes/No*
Q27-1: How often do they take place each week?	$number\ x$
Q28: Do interdisciplinary ward rounds involving pharmacy or	Yes/No

clinical pharmacology take place in your intensive care unit?	
Q29: How often do they take place each week?	$number\ \ x$
Q30: Is a hospital hygienist regularly (at least once a month) present in the intensive care unit?	Yes/No
Q31: How many blood cultures are taken per 1,000 patient days?	$number\ x$
Q32: How many blood culture bottles do you take per collection site?	$number\ x$
Q33: How many different sites do you routinely take blood cultures from per collection?	$number\ x$
Q34: Do you have access to a blood culture cabinet directly in your intensive care unit?	Yes/No
Q35: How are blood culture bottles transported from the intensive care unit to the microbiology laboratory?	$Pneumatic\ tube\ service$<br>Courier service<br>$on\ foot\ in\ house$<br>$other$<br>$no\ answer$
Q36: How long is the average time resulting from the transportation logistics used from the collection of the blood culture to its arrival in your laboratory (e.g. direct pneumatic tube shipment vs. courier service with only 1-3x daily sample collection)?	$\leq 2h$<br>$\leq 6h$<br>$\leq 12h$<br><br>>12h<br>$other$<br>$no\ answer$
Q37: Does your laboratory process blood cultures 24 hours a day?	Yes/No
Q38: Is the blood culture processed in your laboratory 7d/week?	Yes/No
Q39: Does your hospital routinely provide advance information by telephone in the event of a positive blood culture?	Yes/No
Q40: Is "time-to-positivity" reported in your hospital?	Yes/No
Q41: Is a preliminary antibiogram routinely reported in your hospital?	Yes/No
Q42: Is the minimum inhibitory concentration (MIC) routinely reported in your hospital?	Yes/No
Q43: Does your hospital use molecular biological methods (e.g. SeptiFast®, SepsiTest® or similar)	Yes/No

for rapid pathogen identification in the blood of patients with suspected sepsis?	
Q44: Does your hospital participate in one or more Clinical information system (KISS) modules of the National Reference Centre for the Surveillance of Nosocomial Infections?	*Yes/No*
Q44-1: If yes: Which Clinical information system (KISS) modules does your hospital participate in?	*CDAD − KISS*<br>*HAND − KISS*<br>*ITS-KISS*<br>*MRSA − KISS*<br>*NEO − KISS*<br>*ONKO − KISS*<br>*OP − KISS*<br>*STATIONS − KISS*<br>*other*
Q45: Which nosocomial infections are routinely recorded in your intensive care unit as part of infection surveillance, even if you do not have an (ITS) KISS in place?	No systematic recording of nosocomial infections<br>Primary sepsis (incl. catheter − associated infections)<br>Lower respiratory tract infections (incl. VAP)<br>Urinary tract infections<br>(incl. association with transurethral urinary catheters)<br>Meningitis/ventriculitis<br>Other
Q46: Do you keep resistance statistics in your hospital?	*Yes/No*
Q47: Are these resistance statistics reported to the medical management of the intensive care unit at regular intervals?	*Yes/No*
Q47-1: If so, how often are these resistance statistics reported?	1*x/year*<br>2*x/year*<br>*3x/year*<br>4*x/year*<br>> 4*x/year*<br>*no answer*
Q48: Do you have an SOP for "Therapy of sepsis or septic shock"? Shock"?	*Yes/No*
Q48-1: If yes: on which consensus definition is the diagnosis based?	*SEPSIS* − 1<br>*SEPSIS* − 2<br>*SEPSIS-3*<br>*no answer*
Q49: Has an SOP "Blood culture" been established in your intensive care unit?	*Yes/No*
Q50: Do you routinely change CVCs and/or arteries in your intensive care unit?	*Yes/No*

Q51: Does your department hold regular hygiene training courses?	Yes/No
Q52: How often do these hygiene training courses take place?	$less\ than\ 1x/year$<br>$1x/year$<br>$2x/year$<br>$4x/year$<br>$other$<br>$no\ answer$

Table S1: All questions and possible answers in the survey.
ICU; Intensive Care Units, SOFA; Sequential Organ Failure Assessment, SOP; Standard operating procedure, CVC; central venous catheter
